# Triboelectric nanogenerator based on silane-coupled LTA/PDMS for physiological monitoring and biomechanical energy harvesting

**DOI:** 10.1038/s41378-024-00796-0

**Published:** 2024-10-25

**Authors:** Muhammad Umair Khan, Deepa Dumbre, Yawar Abbas, Moh’d Rezeq, Anas Alazzam, Nahla Alamoodi, Maryam Khaleel, Baker Mohammad

**Affiliations:** 1 Department of Computer and Information Engineering, Khalifa University, Abu Dhabi, UAE; 2 System on Chip Lab, Khalifa University, Abu Dhabi, UAE; 3 Department of Chemical Engineering, Khalifa University, Abu Dhabi, UAE; 4 Department of Physics, Khalifa University, Abu Dhabi, UAE; 5 Department of Mechanical Engineering, Khalifa University, Abu Dhabi, UAE; 6 Center for Catalysis and Separations, Khalifa University, Abu Dhabi, UAE; 7 Research and Innovation Center on CO2 and H2, Abu Dhabi, UAE

**Keywords:** Electronic properties and materials, Electronic devices, Electronic properties and materials

## Abstract

Energy harvesting from ambient sources present in the environment is essential to replace traditional energy sources. These strategies can diversify the energy sources, reduce maintenance, lower costs, and provide near-perpetual operation of the devices. In this work, a triboelectric nanogenerator (TENG) based on silane-coupled Linde type A/polydimethylsiloxane (LTA/PDMS) is developed for harsh environmental conditions. The silane-coupled LTA/PDMS-based TENG can produce a high output power density of 42.6 µW/cm^2^ at a load resistance of 10 MΩ and operates at an open-circuit voltage of 120 V and a short-circuit current of 15 µA under a damping frequency of 14 Hz. Furthermore, the device shows ultra-robust and stable cyclic repeatability for more than 30 k cycles. The fabricated TENG is used for the physiological monitoring and charging of commercial capacitors to drive low-power electronic devices. Hence, these results suggest that the silane-coupled LTA/PDMS approach can be used to fabricate ultra-robust TENGs for harsh environmental conditions and also provides an effective path toward wearable self-powered microelectronic devices.

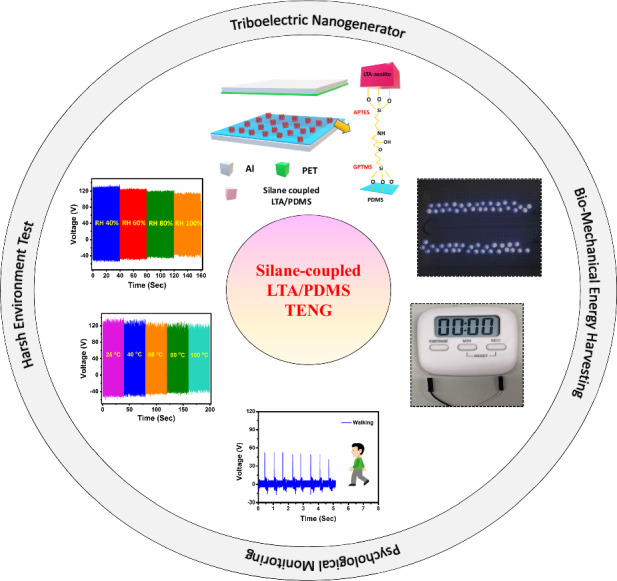

## Introduction

The growth of electronic devices in daily life is increasing rapidly and is expected to improve people’s overall well-being^1–6^. For health care, smart homes, smart cities, autonomous vehicles, and space applications, the need for electronics to sense, compute and communicate is essential. Batteries are the energy source that powers all these devices^7–9^. The battery industry faces challenges in keeping up with device constraints, especially cost, energy density, and maintenance^10–12^. Energy harvesting is one way to address energy constraints, especially for hard-to-reach places, wearable electronics, and biosensing^10,13–18^. Energy harvesting has gained much attention over the past few years because it provides a viable and sustainable solution for addressing global energy crises^19–21^. Various methods of harnessing energy have been developed to convert ambient mechanical energy into electrical stimuli, such as triboelectric^22^, piezoelectric^23^, electrostatic^24^, electromagnetic^25^, and pyroelectric methods^26^. Among them, triboelectric nanogenerators (TENGs) are promising means of harvesting clean and sustainable biomechanical energy. To power microelectronic devices, TENGs can convert wasted mechanical energy to electrical power, which is ubiquitous but frequently wasted in daily life^27^. Triboelectricity works on the basis of the triboelectric and electrostatic effects that can be observed in nature when different materials come into contact^28^. In particular, triboelectricity/contact electrification creates polarized static charges on surfaces in contact. At the same time, electrostatic induction transforms mechanical energy into electrical energy due to changes in potential caused by mechanically agitating separations^29,30^. TENGs offer versatile operation modes and have many advantages, such as low cost, lightweight, broad material availability, and high efficiency, even when used at a low operating frequency^31,32^. There is a wide range of materials at opposite ends of the triboelectric series capable of high performance in TENGs based on their charge affinity^33^.

Moreover, the working environment can significantly affect the performance of devices and impact a TENG’s output, creating new challenges^34–39^. Notably, humidity and temperature, as the two primary environmental variables, have the most significant impacts on TENG performance^36,40–45^. Temperature increases can lead to a noticeable decrease in TENG output due to the Joule heating effect^46^, whereas humid conditions can result in the formation of a water layer on tribolayer surfaces, also contributing to reduced TENG output^47^. Several strategies have been explored to develop humidity-resistant TENGs, including surface functionalization^48^ to induce hydrophobicity, the incorporation of polymer-based composites^49^, the implementation of protective enclosures^36,50^, and the introduction of hierarchical nanostructures to achieve superhydrophobicity^51^. Shaukat et al. reported a highly environmentally stable TENG using MOF-5 enclosed in polyethylene, making it resistant to humidity. Zhou et al. utilized particle lithography to fabricate hierarchical 3D polydimethylsiloxane (PDMS) micro- and nanostructures to achieve an interlayer with a superhydrophobic surface that has self-cleaning properties^51^ and integrated it with a single TENG electrode^51^. Zhang et al. modified the surface roughness and hydrophobicity of polyvinyl toluene by blending it with modified boron nitride and using it as the electropositive tribolayer. Despite these efforts, there are remaining research gaps that need to be filled in terms of the stability of the tribo-layers under high-humidity and high-temperature conditions, as well as the cost-effectiveness and flexibility of the device.

Linde Type A (LTA) zeolite is a synthetic material composed of aluminosilicate that has gained much attention because of its 3D structure, good cation-exchange properties, and excellent thermal and chemical stability^52–54^. Together with the ability to control the porosity of LTA zeolite with a pore size of 4.1 Å and the advantage of having a large surface area^55^, its characteristic electropositive property makes it a promising candidate for the fabrication of TENG^56^. This work introduces a novel technique for developing a superhydrophobic TENG utilizing LTA zeolite-coated PDMS (LTA/PDMS) as the triboelectric layer. The superhydrophobicity was imparted by surface modifications of both LTA zeolite powder and PDMS through a two-silane coupling approach. This approach allows the LTA zeolite powder to be chemically bonded to the PDMS surface, increasing the stability, durability, and electropositivity of the tribolayer. The device exhibited a high output power density of 42.6 µW/cm^2^ at a load resistance of 10 MΩ and operated at an open-circuit voltage of 120 V and a short-circuit current of 15 µA, with an active area of 9 cm^2^ under a damping frequency of 14 Hz. High-efficiency TENGs contribute significantly to the advancement of wearable electronics by enabling self-powered operation, offering a variety of benefits including increased battery life, self-powered wearables, energy harvesting, a reduction in environmental impact, enhanced functionality, a flexible and lightweight design, a monitoring system for health and fitness, a monitoring system for remote environments and challenges, and innovations in wearables.

## Experimental section

Ludox HS-40 colloidal silica (98%, Sigma Aldrich), sodium aluminate (98%, Sigma Aldrich), sodium hydroxide (99%, Merck), and deionized water were used for LTA zeolite synthesis. In addition, 3-aminopropyl-triethoxysilane (APTES) (98%, Alfa Aesar), 3-glycidyloxypropyl-trimethoxysilane (GPTMS) (97%, ACROS ORGANICS), and toluene (>99.9%, Merck) were used for zeolite and PDMS functionalization. PDMS was purchased from Dow Corning Company as a SYLGARD^TM^ 184 silicone elastomer base and a curing agent. A 50-µm thick polyethylene terephthalate (PET) substrate and 2-inch-wide aluminum (Al) tape were purchased from a local store. LTA zeolite was synthesized hydrothermally with a gel composition of 1.8 SiO_2_:1 Al_2_O_3_:12 Na_2_O:214 H_2_O^57,58^, and sodium aluminate powder (1.4 g) was added to deionized water (26.2 g) in a polypropylene bottle and stirred for 10 min at room temperature. Sodium hydroxide (6.38 g) was added to this mixture and stirred for 30 min at room temperature to form a clear solution (Solution 1). Ludox HS-40 colloidal silica solution (2.04 g) was added dropwise into Solution 1 while stirring, after which the solution was continuously stirred for 24 h at room temperature for gelation to occur. The resulting gel was left for crystallization at 60 °C for 5 h. Then, the crystals were separated by centrifugation, washed with deionized water, and dried at 80 °C. PDMS substrates were prepared by mixing SYLGARD^TM^ 184 silicone elastomer base and its curing agent at a 10:1 weight ratio (recommended by the manufacturer) in a polypropylene boat for 10 min, followed by degassing in a vacuum desiccator for 30 min (until the bubbles disappeared). This mixture was poured onto a clean and dry silicon wafer and cured on a hotplate at 60 °C for 4 h. The PDMS slab was then peeled from the silicon wafer and cut into small pieces of 4 cm^2^, 9 cm^2^, and 16 cm^2^ for all the experiments. To fabricate the triboelectric layer, the as-synthesized LTA zeolite powder (0.2 g) was added to a mixture of APTES (0.2 M) in toluene (10 mL) at 110 °C for 1 h (Supplementary Fig. S1). Next, the solution was centrifuged, and the LTA crystals were washed three times with a toluene and ethanol solution to remove the unreacted silane, followed by drying overnight at 80 °C. Prior to attaching the functionalized zeolites to PDMS, the PDMS substrate was first activated by plasma treatment for 5 min at 626 mTorr and 1–2 scfh of oxygen with 29.6 W of power and a radiofrequency of 12 MHz to increase the concentration of the polar OH groups^59^ on the surface and then silanized with GPTMS. For silanization with GPTMS, the substrate was immersed in a solution of GPTMS (0.2 M) in toluene (10 mL) at 110 °C for 1 h. This GPTMS-treated PDMS substrate was dipped into a suspension of APTES-treated LTA zeolite in toluene (0.025 g, 0.050 g, or 0.1 g in 10 mL of toluene) at 110 °C for 1 h. The LTA-coated PDMS substrate was removed from the reaction mixture, washed extensively with toluene, and dried at 80 °C for 24 h.

## Results and discussion

**Fig. 1:**
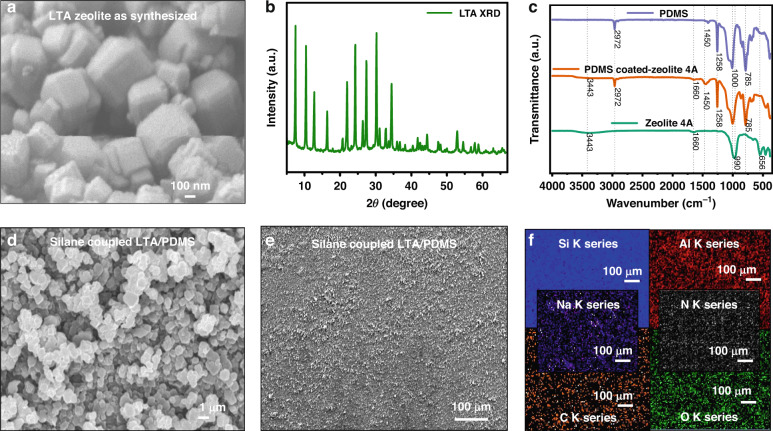
Material characterization of LTA zeolite and silane-coupled LTA/PDMS. **a** SEM image and **b** XRD pattern of the as-synthesized LTA zeolite. **c** FTIR spectra of the as-synthesized LTA-zeolite, PDMS, and silane-coupled LTA/PDMS. **d** SEM images of the LTA zeolite-coated PDMS substrate at 1 µm and **e** a magnified SEM image at 100 µm. **f** EDX color map of the LTA/PDMS electropositive layer showing Si, Al, Na, N, C, and O

The details of the chemical characterization are discussed in Supplementary Section 2. Furthermore, Supplementary Fig. S1 illustrates a two-component modification strategy, which mainly led to the assembly of APTES-tethered LTA zeolite on the GPTMS-coated PDMS substrate. This approach involves independent tethering of two functional groups, namely, amine and epoxy functional groups, on LTA zeolite powder and the PDMS substrate, respectively, followed by covalent linking of both functional groups to form the final product assembly (Supplementary Fig. S1). The SEM image (Fig. 1a) shows LTA zeolite cubic particles with sizes ranging between 250 nm and 300 nm. The successful synthesis of LTA zeolite with high purity and high crystallinity was confirmed via X-ray diffraction (XRD) (Fig. 1b), where all the peaks were indexed as cubic LTA (by comparison with standard LTA JCPDS (37–1233) spectra). The FTIR spectra of the as-synthesized LTA, PDMS, and silane-coupled LTA/PDMS electropositive layers are shown in Fig. 1c. The FTIR spectra confirmed the presence of the LTA zeolite coating on the PDMS surface, where –OH stretching and bending vibrations were observed at 3443 cm^−1^ and 1660 cm^−1^, respectively. The vibration peaks at 990 cm^−1^, 656 cm^−1^, and 459 cm^−1,^ which are assigned to the stretching, bending, and rocking vibrations of T–O–T bridges (T = Si, Al), respectively, can be observed for both as-synthesized LTA and LTA-coated PDMS^60,61^. In addition, peaks at 785 cm^−1^ and 1258 cm^−1^ for -Si-CH3- stretching and bending vibrations, respectively, are also observed for PDMS and PDMS-coated zeolite 4 A^62^. The peak at 1000 cm^−^^1^ corresponds to Si-O-Si stretching vibrations, whereas 1450 cm^−^^1^ and 2972 cm^−^^1^ vibrations were observed for asymmetric methyl stretching in Si–CH_3_ for both the bare PDMS and LTA-coated PDMS samples. Bonds containing nitrogen (N) were not detected by FTIR, which could be a result of the minute amount of N-containing functional groups and the orientation of the bonds. The assembled silane-coupled LTA/PDMS substrate is used as electropositive layer for the fabrication of TENG device. SEM images (Fig. 1d) revealed that the PDMS substrates were completely covered by LTA-zeolite crystals to a scale of less than 1 µm with multiscale porosity and no visible cracks even after washing with toluene. The SEM image of bare PDMS (Supplementary Fig. S2) shows a smooth surface. In contrast, the SEM image of GPTMS-treated PDMS without zeolite (control experiment) shows a wrinkled surface morphology due to plasma treatment (Supplementary Fig. S2). Contact angle measurements of PDMS (bare), PDMS-treated O2-plasma, GPTMS-anchored PDMS, and GPTMS (2 mM) + APTES (2 mM) are depicted in Supplementary Fig. S3a–d. The amount of deposited zeolite crystals depends on the initial amount of dispersed LTA zeolite (Supplementary Fig. S3e–g) during synthesis; since quantifying the amount of deposited crystals is difficult, we focused on the coverage of the samples as an indication of the completion of the coating process. Hence, EDX spectra were analyzed for APTES-functionalized LTA zeolite crystals compared with unmodified zeolite crystals (Supplementary Fig. S4), and the results confirmed the presence of nitrogen in the functionalized zeolites and its absence for LTA zeolites. The elemental EDS color map of the LTA/PDMS electropositive layer at 100 µm (Fig. 1e) confirmed the amounts of various elements present, such as Si, Na, C, O, N, and Al, as shown in Fig. 1f. The average thickness of the LTA zeolite coating is ~10.4 µm, and the overall thickness of the LTA/PDMS substrate is ~0.2 mm.

**Fig. 2:**
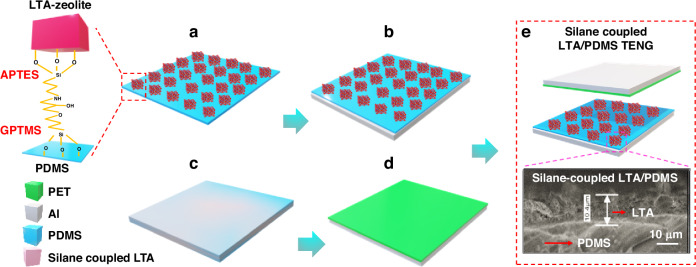
The fabrication process of the silane-coupled LTA/PDMS TENG device. **a**–**d** The silane-coupled LTA/PDMS TENG preparation process and **e** device schematic stacked structure and cross-sectional image of the silane-coupled LTA/PDMS electropositive layer

The device fabrication steps are shown in Fig. 2. Conductive adhesive Al tape was used as the electrode for charge collection. The silane-coupled LTA/PDMS (as the electropositive layer) and PET (as the electronegative layer) substrates were fixed on the adhesive side of the Al tape, and copper wires were affixed to the backside of the electrodes. The dimensions of the silane-coupled LTA/PDMS TENG device were 4 cm^2^, 9 cm^2^, and 16 cm^2^, and the spacing between the electropositive and electronegative layers was maintained at 4 mm. The device structure of the silane-coupled LTA/PDMS TENG is schematically shown in Fig. 2a–e. The average thickness of the silane-coupled LTA coating is ~10 µm, as shown in Fig. 2e. The cross-sectional view clearly shows the uniform deposition and consistency of the coating across the substrate surface, and the overall thickness of the silane-coupled LTA/PDMS layer is ~0.2 mm. The experimental setup used for device testing is shown in Supplementary Fig. S5.

**Fig. 3:**
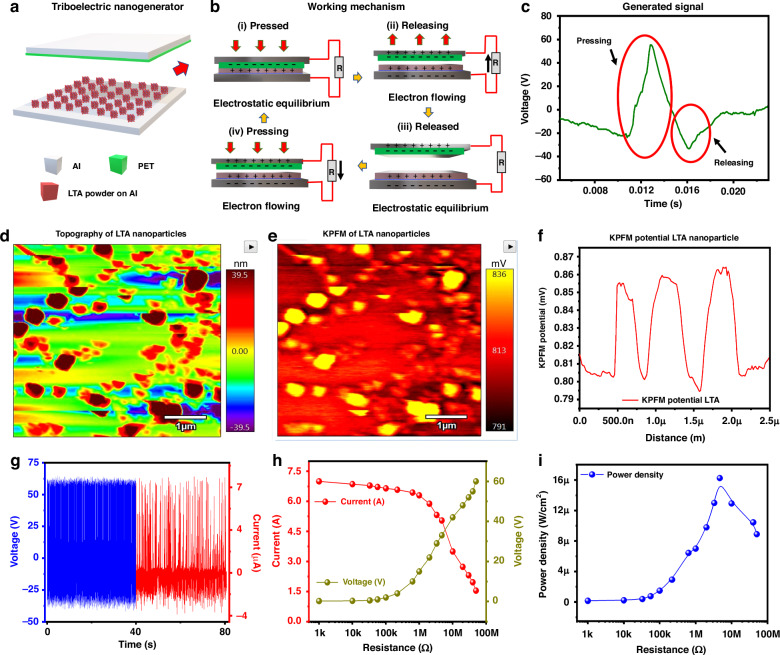
Electrical characterization and working mechanism of the LTA zeolite TENG device. **a** Schematic illustrating the TENG using as-synthesized LTA zeolite powder. **b** Charge generation and transfer mechanism of the TENG. **c** Generated output voltage signal of the TENG. **d** Topography of LTA zeolite nanoparticles. **e** KPFM results showing the positive potential of the LTA zeolite nanoparticles. **f** KPFM potential of LTA zeolite nanoparticles. **g** The open-circuit voltage and short-circuit current of the TENG were measured using LTA zeolite powder. **h** Loading effect on the output current and voltage response. **i** Power density with variable load resistance from 1 kΩ to 50 MΩ

Initially, synthesized LTA zeolite powder rubbed on an Al substrate was used to fabricate the TENG (Al/PET/(spacer)/LTA zeolite (powder)/Al), as shown in Fig. 3a. This device structure helps in understanding the device’s performance and mechanism. The working mechanism of the LTA zeolite TENG device is shown in Fig. 3b. The LTA zeolite layer is positively charged, whereas the surface of the polyethylene terephthalate (PET) layer is negatively charged, as shown in Fig. 3b(i). An external force must be applied to the device to establish contact between the LTA zeolite powder as an electropositive layer and PET as an electronegative layer, as shown in Fig. 3b(i). The difference between the electron affinities of the two tribomaterials as they contact each other results in electron donation from the oxygen atoms of the LTA zeolite to the PET layer. Upon removal of the external force, free electrons flow from the upper electrode (PET electrode) to the bottom electrode (LTA zeolite electrode), and induced charges are generated on both electrodes, producing an electric current, as shown in Fig. 3b(ii). The generated signal while pressing is reflected in Fig. 3c. The electrons continue to flow until the external force is completely withdrawn; at this point, the current stops, as illustrated in Fig. 3b(iii). When the two tribolayers are in contact again under the influence of an external force, the external circuit experiences a reverse current flow because the induced electrons return, as shown in Fig. 3b(iv). The reverse current during release is shown in Fig. 3c. As a result, when this cycle continues, an ongoing alternating current is produced.

The dimension (area) of the as-synthesized LTA powder rubbed on the Al-tape TENG device is 9 cm^2^. The electropositive nature of the zeolite nanoparticles was investigated via the Kelvin probe force microscopy (KPFM) technique, as shown in Fig. 3d–f. During KPFM analysis, the tip scans twice over the sample, where the first scan produces the topography, and the second scan measures the potential on the surface. The accurate potential of a sample depends upon the electrical tuning of the conducting tip used for the analysis. The optimized potential, which produced accurate surface potential mapping in this experiment, was 2.35 V. The topographic nature of the LTA zeolite nanoparticles resulted in a surface roughness of 39.5 nm, as shown in Fig. 3d. The KPFM results of the zeolite nanoparticles show a positive potential of 800 mV, as shown in Fig. 3f. The TENG electrical characterisation steps are discussed in Supplementry Section 6 and TENG characterisation setup is shown in Supplementry Fig. S5. The open-circuit voltage was recorded as ~60 V, and the short-circuit current reached 7 µA, with a damping frequency of 14 Hz, as shown in Fig. 3g. When the synthesized LTA zeolite powder TENG was connected to the external load resistance range of 100 kΩ to 50 MΩ, the output voltage increased, whereas the current decreased with increasing loading resistance, as shown in Fig. 3h. The output power density was calculated to be 16.2 µW/cm^2^ at a load resistance of 5.7 MΩ, as shown in Fig. 3i.

**Fig. 4:**
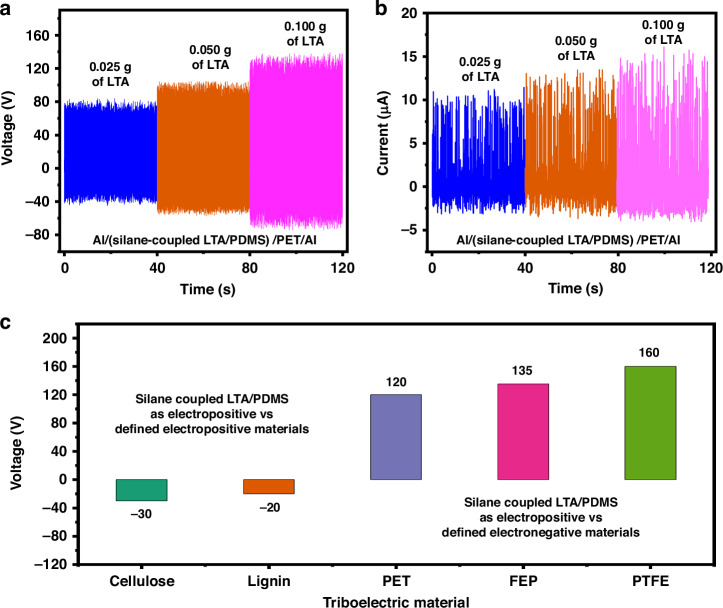
Effect of different cross-linking concentrations of LTA on PDMS and performance comparison of silane-coupled LTA/PDMS with various electropositive and electronegative materials. **a** open-circuit voltage and **b** short-circuit current of different concentrations of silane coupled LTA/PDMS. **c** Silane-coupled LTA/PDMS performance compared with that of different electropositive and electronegative materials

The results depicted in Fig. 3g–i necessitate improving the open-circuit voltage, short-circuit current, and power density. The device was then modified by anchoring the LTA zeolite powder to a thin film of PDMS via the two-silane coupling strategy. The PDMS layer must be fabricated into a thin film to ensure the flexibility of the electropositive layer. The silane-coupled LTA/PDMS composite possesses two main characteristics: high charge electrification; the hydroxyl groups from LTA zeolite play a dominant role in LTA zeolite as oxygen-based functional groups with a significant electron-donating potential effect during the triboelectrification process and high water resistance; and the PDMS and cross-linkers (APTES and GPTMS) heavily contribute to the superhydrophobic behavior of the LTA zeolite crystals, which ultimately enhances TENG performance under harsh environmental conditions with stable and reproducible performance. Both of these characteristics are further confirmed in subsequent studies. LTA zeolite coverage on the PDMS surface can be varied by changing the amount of APTES-functionalized LTA powder added during the crosslinking step with GPTMS/PDMS. As shown in Fig. 4a, b, the output of the TENG performance changes with the content of LTA-zeolite coated on the PDMS substrate such that LTA zeolite loadings of 0.1 g, 0.05 g and 0.025 g result in open-circuit voltages of 120 V, 100 V and 80 V, respectively, and currents of 15 µA, 13 µA, and 11 µA, respectively. As expected, a higher LTA zeolite content results in greater performance in the TENG device because of the increased electropositive nature of the LTA/PDMS layer, as indicated above; this is well aligned with the SEM images shown in Supplementary Fig. S3e–g. Highly uniform and high-density close-packing coverage of LTA zeolite crystals on the entire PDMS surface for 0.1 g LTA-zeolite content (Supplementary Fig. S3g) resulted in better TENG performance. The lower TENG performance observed for 0.025 g and 0.050 g (Supplementary Fig. S3e, f) LTA-zeolite mixtures is attributed to less coverage of LTA-zeolite crystals on the PDMS surface. Notably, for the sizes used in this study, 0.1 g of LTA zeolite should be sufficient to achieve enhanced TENG performance. Figure 4c shows the analysis of the contact electrification capability of the silane-coupled LTA/PDMS TENG with various electropositive materials (cellulose, lignin) and electronegative materials (PET, fluoroethylene propylene (FEP), and polytetrafluoroethylene (PTFE)). The electron-donating or electron-attracting ability of a material determines the voltage amplitude. According to the triboelectric series, PTFE can draw electrons from the other contacted surface, resulting in high voltage, as illustrated in Fig. 4c. On the other hand, compared with FEP and PTFE, PET has a modest electron-attracting capacity. The electrostatic induction of silane-linked LTA/PDMS corresponds well with the triboelectric series, indicating that the silane-coupled LTA/PDMS electropositive layer has a substantial electron-donating capacity during triboelectrification.

**Fig. 5:**
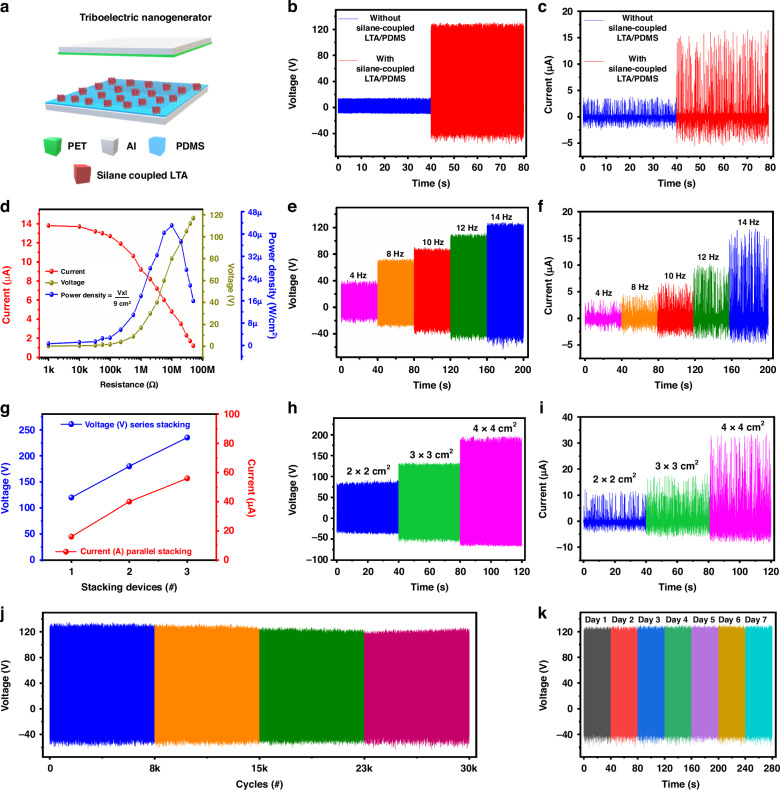
Electrical characterisation of the silane-coupled LTA/PDMS TENG device. **a** Schematic illustration of the TENG using silane-coupled LTA/PDMS. **b** Open-circuit voltage and **c** short-circuit current of the TENG without and with silane coupling of LTA/PDMS. **d** Output current, voltage and power density with variable load resistance from 1 kΩ to 50 MΩ. **e** Open-circuit voltage and **f** short-circuit current at different frequency levels in the ranges of 4 Hz, 8 Hz, 10 Hz, 12 Hz, and 14 Hz. **g** Stacking of 1, 2, and 3 units of the LTA/PDMS TENG for voltage measurement in series and for current measurement in parallel. The effect of device size (4 cm^2^, 9 cm^2^ and 16 cm^2^) on device performance is presented, showing the **h** open-circuit voltage and **i** short-circuit current. **j** Stability performance of the silane-coupled LTA/PDMS TENG for 30 k cycles. **k** TENG device stability was analyzed for 7 days

A schematic illustration of the optimized TENG using silane-coupled LTA/PDMS at 0.1 g, which was further characterized for detailed device (Al/PET/(spacer)/silane-coupled LTA/PDMS/Al) analysis, is shown in Fig. 5a. The open-circuit voltage was recorded as ~120 V, as shown in Fig. 5b, and the short-circuit current reached 15 µA, as shown in Fig. 5c, demonstrating that the electrical performance of the silane-coupled LTA/PDMS (loading 0.1 g of LTA zeolite) TENG is significantly greater than that of the powder based LTA zeolite TENG, as shown in Fig. 3g–i. To confirm the electropositive nature of the silane-coupled LTA/PMDS, the analysis was repeated without the silane coupling of the LTA/PMS zeolite layer with the device structure of Al/PET/(spacer)/Al. The device output performance reached 16 V and 3 µA, as illustrated in Fig. 5b, c. These results prove that the high charge electrification is due to the silane coupling of the LTA/PDMS layer. To further ensure that the output generated was from the TENG rather than from noise or external sources, a polarity switching test of the silane-coupled LTA/PDMS TENG is shown in Supplementary Fig. S6. The instantaneous power of the LTA/PDMS TENG was also studied, which was determined by increasing the load resistance from 1 kΩ to 50 MΩ, as shown in Fig. 5d. As the load resistance increases from 1 kΩ to 50 MΩ, the current decreases, and the open-circuit voltage increases. When the input resistance equals the load resistance, the TENG can generate a maximum power density of 42.6 µW/cm^2^ at a load resistance of 10 MΩ, as shown in Fig. 5d. Moreover, the TENG output performance was analyzed using different frequency ranges of 4 Hz, 8 Hz, 10 Hz, 12 Hz, and 14 Hz, as shown in Fig. 5e, f. The open-circuit output voltage varies from ~40 V to ~120 V, and the short-circuit current varies from 3 µA to 15 µA. When the frequency is decreased from 14 Hz to 4 Hz, the output performance of the TENG decreases. The frequency of contact influences the number of contact and separation cycles per unit of time. There are fewer contact and separation cycles per unit of time when the frequency of contact is low, which may result in a lower voltage output. When the contact frequency is high, there are more contact and separation cycles per unit of time, which might result in a greater voltage output. Depending on the application, a single nanogenerator may not have sufficient power for specific devices. As a result, several researchers have proposed novel techniques to achieve higher energy by integrating more TENGs into the same instrument^63,64^. Multiple stacking units are connected in series or parallel to achieve significant energy. A series of connections increase the voltage, and parallel connections increase the driving current. Figure 5g shows the output performance of the stacked silane-coupled LTA/PDMS TENG in both parallel and series configurations; when 1, 2, or 3 units are connected in series, the open-circuit voltage increases in increasing order to 120 V, 160 V, and 220 V, respectively. Similarly, estimated short-circuit currents of 15 µA, 38 µA, and 56 µA were achieved by connecting 1, 2, and 3 units in the parallel circuit, respectively. The stability of two and three TENGs stacked in series has been proven for more than 14 k cycles, as shown in Supplementary Fig. S7. As a result, by simply designing various stacking units, the output performance of the silane-coupled LTA/PDMS TENG may be increased to the required value, making the device more practical for large-scale commercial applications. Figure 5h, i shows the effects of device size optimization on the electrical performance of the proposed LTA/PDMS TENG. The device sizes used were 4 cm^2^, 9 cm^2^, and 16 cm^2^. The 4 cm^2^ device revealed a potential of 75 V and a current of 10 µA, whereas the 9 cm^2^ device showed a potential of 120 V and a current of 15 µA. After extending the device size to 16 cm^2^, the TENG obtained greater voltage (180 V) and current (32 µA) values. A greater surface contact area between each device improved its performance while increasing its size. A linear damping motor was used to analyze the stability of the silane-coupled LTA/PDMS TENG. As shown in Fig. 5j, the LTA/PDMS TENG operates over 30 k cycles, demonstrating a high level of stability and endurance performance in the frequency range of 14 Hz. Furthermore, the device showed long-term stability, robustness, and endurance for over 7 days, as illustrated in Fig. 5k. The successful cycling test underscores the viability of the TENG for integration into wearable electronics, health monitoring devices, and reliable energy harvesting from human activities under harsh environmental conditions. These results indicate that the fabricated TENG can open the door to reliable, high-performance energy harvesting applications.

### Application

**Fig. 6:**
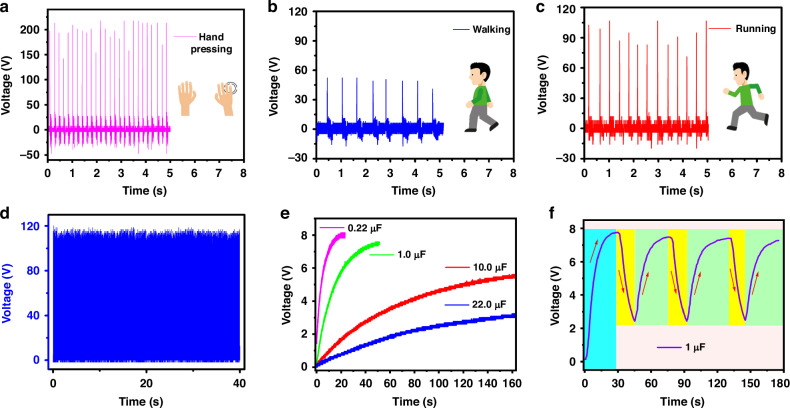
Monitoring of human motion states and capacitor charging for powering wearable electronic devices. **a** Hand pressing, **b** walking, and **c** running. **d** The rectified voltage response of the silane-coupled LTA/PDMS TENG. **e** Charging characteristics of various capacitors (0.22 µF, 1.0 µF, 10 µF, and 22 µF) and **f** the charging and discharging cycles of a 1-µF commercial capacitor

The LTA/PDMS TENG may be utilized for energy harvesting and detecting mechanical motions. TENGs hold paramount importance in hand-tapping, walking, and running scenarios because of their capacity to convert mechanical energy from these human motions into electrical energy. In hand tapping, TENGs can be integrated into wearable devices, capturing energy generated by finger taps or gestures to power small electronic components, offering a convenient and sustainable energy source for interactive devices. The adaptability of TENG technology to these activities addresses the growing demand for efficient and portable energy sources, enhancing the feasibility of self-powered electronic devices integrated seamlessly into daily human activities. The hand tapping of the TENG was monitored with an output voltage of 200 V, as shown in Fig. 6a. For walking and running, TENGs embedded in shoes or clothing can harness the kinetic energy produced during each step, providing a self-sufficient power supply for various applications, including wearable health monitors, activity trackers, and communication devices. The device is highly sensitive to human body movement and can easily detect an output signal for monitoring. A silane-coupled LTA/PDMS TENG was mounted on the bottom of the shoes to monitor various motion states of the human body, such as walking and running, as presented here. The device’s peak output voltage while walking was approximately 55 V, as shown in Fig. 6b. The two friction layers in the TENG moved toward one another and eventually made contact to produce a peak voltage as soon as the foot touched the ground. The two friction layers split apart as the foot lifts, creating an opposing voltage. While running, due to the increased pressure placed on the device, as illustrated in Fig. 6c greater peak voltage of approximately 105 V was observed during operation. In addition to harvesting energy from body movements, the results show that the silane-coupled LTA/PDMS TENG can record moving posture and that the characteristics of the output signals can be used to analyze an athlete’s precise body movements; this shows the great potential of our device for use in sports and health. The rectified output voltage of the silane-coupled LTA/PDMS TENG, as shown in Fig. 6d, was used to operate low-power devices. A detailed analysis of the rectified signal is shown in Supplementary Fig. S8. Using continuous stepping, the TENG was evenly pressed to charge various capacitors using 10 MΩ impedance probe at 14 Hz for 160 s to study its charging properties. Figure 6e shows the response of the capacitors in the range of 0.22–22 µF, indicating a quick charging rate with a maximum voltage of 8 V by 0.22 µF. In addition, Fig. 6f depicts the stable charging and discharging cycles of a 1-µF capacitor up to 7.8 V. TENGs offer much potential for harvesting biomechanical energy since they can efficiently transform mechanical energy from human body movements into electrical energy. TENGs can power wearable devices such as stopwatches, calculators, and LEDs by harvesting energy from body motions, thereby minimizing external power source requirements, as shown in Supplementary Fig. S9.

**Fig. 7:**
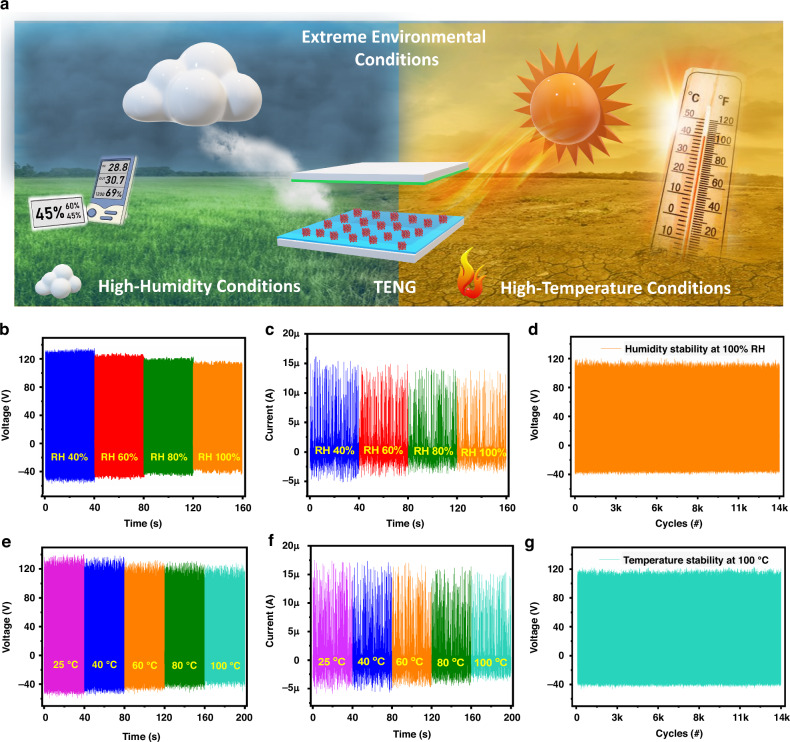
Performance of TENG under extreme environmental conditions. **a** Schematic demonstrating the extreme environmental conditions for TENGs. The humidity test of the TENG device at RHs of 40%, 60%, 80%, and 100% shows the **b** open-circuit voltage and **c** short-circuit current. **d** Humidity stability at 100% RH. TENG performance at different temperatures in the ranges of 25 °C, 40 °C, 60 °C, 80 °C, and 100 °C, showing **e** the open-circuit voltage and **f** short-circuit current. **g** Temperature stability at 100 °C

Environmental variables such as temperature and humidity significantly impact TENG output performance, as shown in Fig. 7a. Hence, the influences of humidity and temperature on the tribolayers of the fabricated TENG were investigated. The TENG performance was tested at different humidity levels: 40%, 60%, 80%, and 100% RH at room temperature (25 °C). The slight drop in performance at 40%, 60%, 80%, and 100% relative humidity produces open-circuit voltages of 100%, 91.06%, 89.55%, and 85.82%, respectively, compared with those of TENGs operated under ambient conditions, as shown in Fig. 7b. Similarly, 40%, 60%, 80%, and 100% RH short-circuit currents produce outputs of 100%, 96%, 92.8% and 89.5%, respectively, as shown in Fig. 7c. The slight drop in output (open-circuit voltage and short-circuit current) could be attributed to water molecules accumulating on the PET surface, which has a contact angle 78.9. The slight drop in the output performance of the TENG results from the superhydrophobicity of the LTA/PDMS electropositive layer, which contrasts with the highly hygroscopic nature of LTA zeolites. The device stability was analyzed at 100% RH for more than 14 k cycles, which revealed no signs of deterioration under highly humid conditions, as shown in Fig. 7d. Furthermore, the device was tested at 25 °C, 40 °C, 60 °C, 80 °C, and 100 °C at 40% RH. The highest temperature selected mimics harsh environments for the physiological and biomechanical energy harvesting applications considered in this work. The results show relatively constant output open-circuit voltages of 100%, 98.50%, 94.02%, 92.53%, and 91.04%, respectively, as shown in Fig. 7e, and output short-circuit currents of 100%, 99.4%, 97.1%, 92.5% and 87.35%, respectively, as shown in Fig. 7f, demonstrating that a temperature change has a minor effect on the output performance. LTA zeolite is known for its high thermal resistance, which also minimizes the impact of thermal expansion and contraction, contributing to the material’s stability over a temperature range of 25 °C to 100 °C and preventing agglomeration even under extreme temperatures. Consequently, the tribopositive film in the TENG exhibits reliable and sustained performance, making it suitable for energy harvesting applications in environments characterized by fluctuating temperatures. The device stability for more than 14 k cycles at 100 °C indicates that the TENG is highly robust and environmentally stable under high-temperature conditions, as shown in Fig. 7g.

### Concluding remarks

We have successfully developed a high-performance and environmentally robust TENG using a surface coating of LTA zeolite on PDMS via a two-silane anchoring strategy. The TENG was fabricated using silane-coupled LTA/PDMS and PET as the triboelectric layers. The device with an active area of 9 cm^2^ generated an open-circuit voltage of 120 V and a short-circuit current of 15 µA at 14 Hz. A power density of 42.6 µW/cm^2^ at a 10-MΩ load resistance was obtained. The operating frequency is suitable for human activities, which makes this suitable for wearable electronics. Moreover, the performance of TENGs is highly stable with respect to electrical output under harsh environmental conditions (humidity and temperature). The endurance performance of the TENG was recorded for more than 30,000 cycles, and retention was monitored for seven continuous days, with stable output performance. The high sensitivity of TENGs is further used to monitor human motion (walking, running, and hand tapping). The ability of the fabricated TENG to charge capacitors to power electronic devices (calculators and stopwatches) was also demonstrated. The TENG was also used to illuminate 56 LEDs connected in a series configuration. Furthermore, a literature summary of the electrical performance of previously reported TENGs and a comparison with a (silane-coupled LTA/PDMS) TENG device are shown in Supplementary Table S1. These results show that the silane-coupled LTA/PDMS TENG is environmentally stable and has the potential to be used for energy harvesting to support wearable electronics and physiological monitoring applications.

## Supplementary information

Supplemental Material File #1

## Data Availability

The datasets used and/or analyzed during the current study are available from the corresponding author upon reasonable request.
