# Supplementary material for: Triboelectric nanogenerator based on silane-coupled LTA/PDMS for physiological monitoring and biomechanical energy harvesting
Source: Microsyst Nanoeng. 2024 Oct 25;10:152. doi: 10.1038/s41378-024-00796-0 (PMC11502885; doi:10.1038/s41378-024-00796-0)
Supplement: Supplementary file 1 — Supplemental Material File #1 [file 41378_2024_796_MOESM1_ESM.docx]

**Supplementary:** **Triboelectric Nanogenerator Based on Silane-coupled LTA/PDMS for Physiological Monitoring and Biomechanical Energy Harvesting**

Muhammad Umair Khan^1,2,⸸^, Deepa Dumbre^3,⸸^, Yawar Abbas^4,2^, Moh'd Rezeq^4,2^, Anas Alazzam^5,2^, Nahla Alamoodi^3,6^*, Maryam Khaleel^3,7,^*, and Baker Mohammad^1,2,^*

^1^Department of Computer and Communication Engineering, Khalifa University, Abu Dhabi 127788, UAE

^2^System on Chip Lab, Khalifa University, Abu Dhabi 127788, UAE

^3^Department of Chemical Engineering, Khalifa University, Abu Dhabi 127788, UAE

^4^Department of Physics, Khalifa University, Abu Dhabi 127788, UAE

^5^Department of Mechanical Engineering, Khalifa University, Abu Dhabi 127788, UAE

^6^Center for Catalysis and Separations, Khalifa University, Abu Dhabi 127788, UAE

^7^Research and Innovation Center on CO_2_ and H_2_, Abu Dhabi 127788, UAE

^⸸^Both authors are considered first and equal contributors to this work.

^*^E-mail: [Nahla.alamoodi@ku.ac.ae](mailto:Nahla.alamoodi@ku.ac.ae), [Maryam.Khaleel@ku.ac.ae](mailto:Maryam.Khaleel@ku.ac.ae), [baker.mohammad@ku.ac.ae](mailto:baker.mohammad@ku.ac.ae)

1. **Synthesis of Silane Coupled LTA/PDMS:**

**
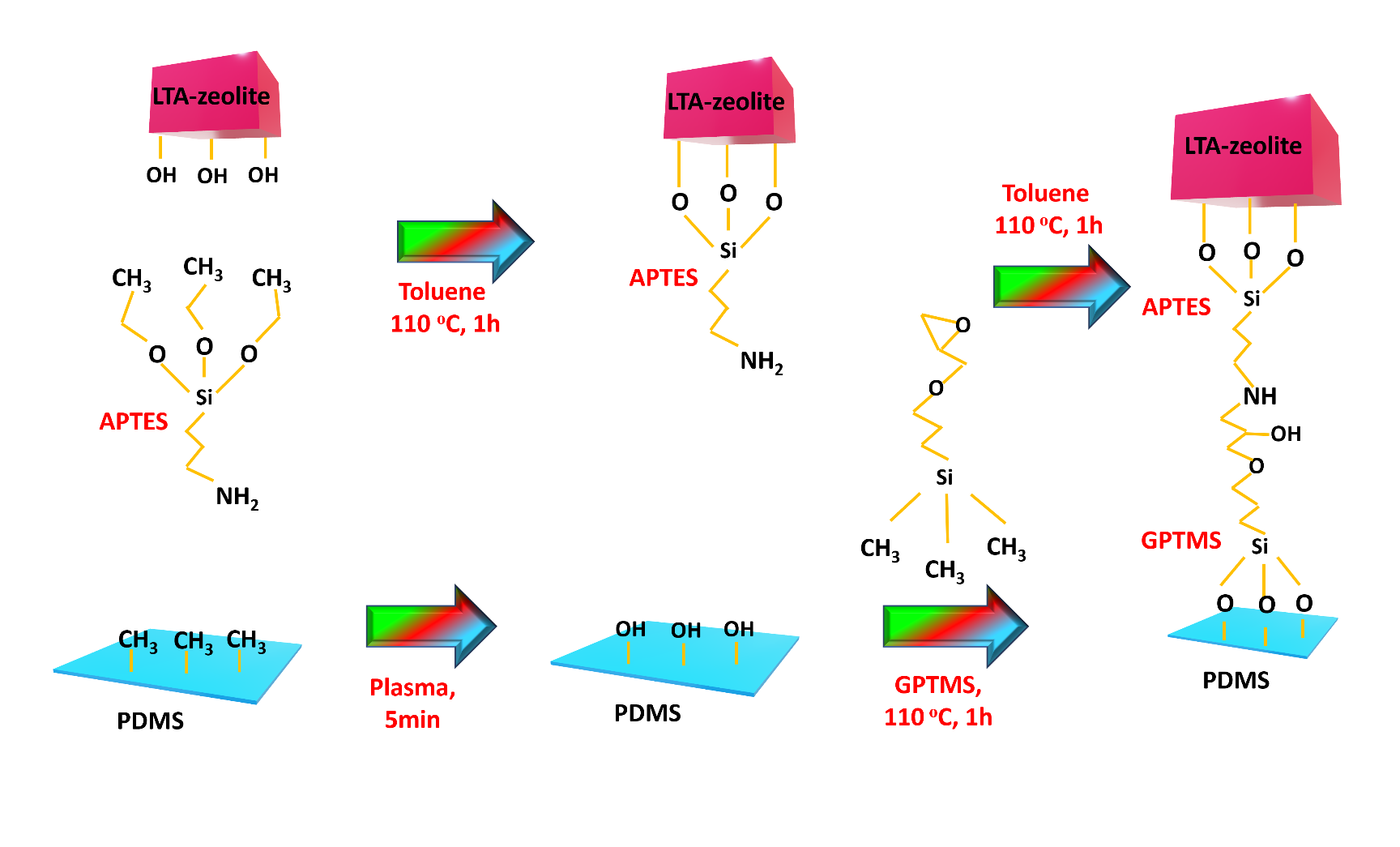
**

**Figure S1:** Schematic illustrating the approach to attach LTA crystals onto PDMS substrate through surface-tethered amino-propyl & epoxy-propyl groups.

1. **Chemical Characterization**

X-ray Diffraction (XRD) was performed using Bruker D8 diffractometer (CuKα radiation) with 2θ range 5^∘^ - 70^∘^ to study the crystallinity of the LTA coating. Fourier Transform Infrared (FTIR) spectroscopy was performed on a Bruker Alpha system equipped with a diamond attenuated total reflectance (ATR) accessory over a wavelength range of 4000 cm^-1^ - 400 cm^-1^ to study the functional groups for as-synthesized LTA zeolite and LTA-coated-PDMS substrates. Scanning Electron Microscopy (SEM) images for all samples were collected using a Qunta 250 SEM machine (JEOL-SEM) with a Schottky field emission gun as an electron source at 10 kV. For SEM, samples were coated using a gold sputter. Elemental analysis for all samples was done using the Apollo silicon drift detector (SDD) integrated with Energy-Dispersive X-ray (EDX) spectroscopy at 10 kV using the Genesis software. A contact angle goniometer (Ossila –L2004A1) was used to measure the water contact angle of as-synthesized LTA zeolite and LTA-coated PDMS substrates at room temperature. The Kelvin Probe Force Microscopy (KPFM) of zeolites was carried out using an Ayslum MFP-3D atomic force microscope (AFM). A conducting gold-coated tip was used to find the potential of the sample. For the KFPM technique, the bias is applied to the conducting tip, and the sample is grounded. The known bias voltage applied to the tip produces an electrostatic force between the tip and sample, which appears as potential on the sample during the potential topography ^1^. The high-frequency silicon tip with an apex diameter of 30 nm was used for imaging. The resonance frequency of the tip is around 256 kHz. The thickness of PDMS is measured using a vernier calliper.

1. **Material Characterization:**

**
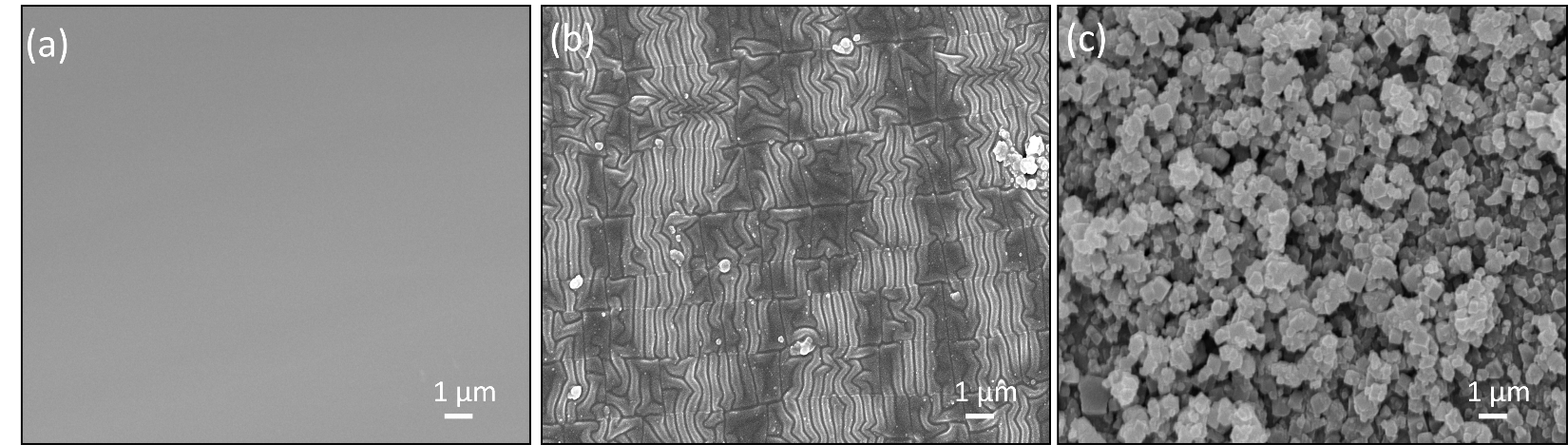
**

**Figure S2:** SEM images of (b) bare PDMS, (b) GPTMS-treated PDMS without zeolite, and (c) LTA-zeolite-coated PDMS.

1. **Contact angle analysis and SEM of Silane Coupled LTA PDMS.**

The surface hydrophobicity was studied using water contact angle measurements. Figure S3a-d shows the water contact angles for PDMS (bare), plasma-treated PDMS, GPTMS-treated PDMS surface, and LTA/PDMS electropositive layer. Being hydrophobic, the bare PDMS surface has a contact angle of 117o, which significantly decreases to 10o upon plasma treatment, as shown in Figure S3b. This decrease results from the highly reactive polar functional groups, mainly the silanol group (Si-OH), at the expense of methyl groups (–CH_3_). A similar result is seen in Figure S3c for the GPTMS-treated PDMS surface, which showed good hydrophilicity with an initial contact angle value of 30.83^o^. Due to the nature of the PDMS, this contact angle increases with time until it reaches the same value as bare PDMS due to hydrophobic recovery. Figure S3d is the contact angle measurement of the LTA/PDMS electropositive layer, showing a value of 155^o^. This superhydrophobic behaviour is attributed to the crosslinking effect of both APTES and GPTMS for anchoring LTA zeolites on PDMS, as well as the multiscale roughness resulting from the deposition of the non-uniform multilayers of LTA zeolite, which are evident from the SEM images, that stabilizes the sliding angle of the surface^2^. The effect on the PDMS surface coverage with initial zeolite content of 0.025 g, 0.050 g, and 0.1 g in the toluene suspension is shown in Figure S3e-g. It can be seen that using an initial amount of LTA zeolite less than 0.1 g results in a thin multi-layered coating of the PDMS surface.

**
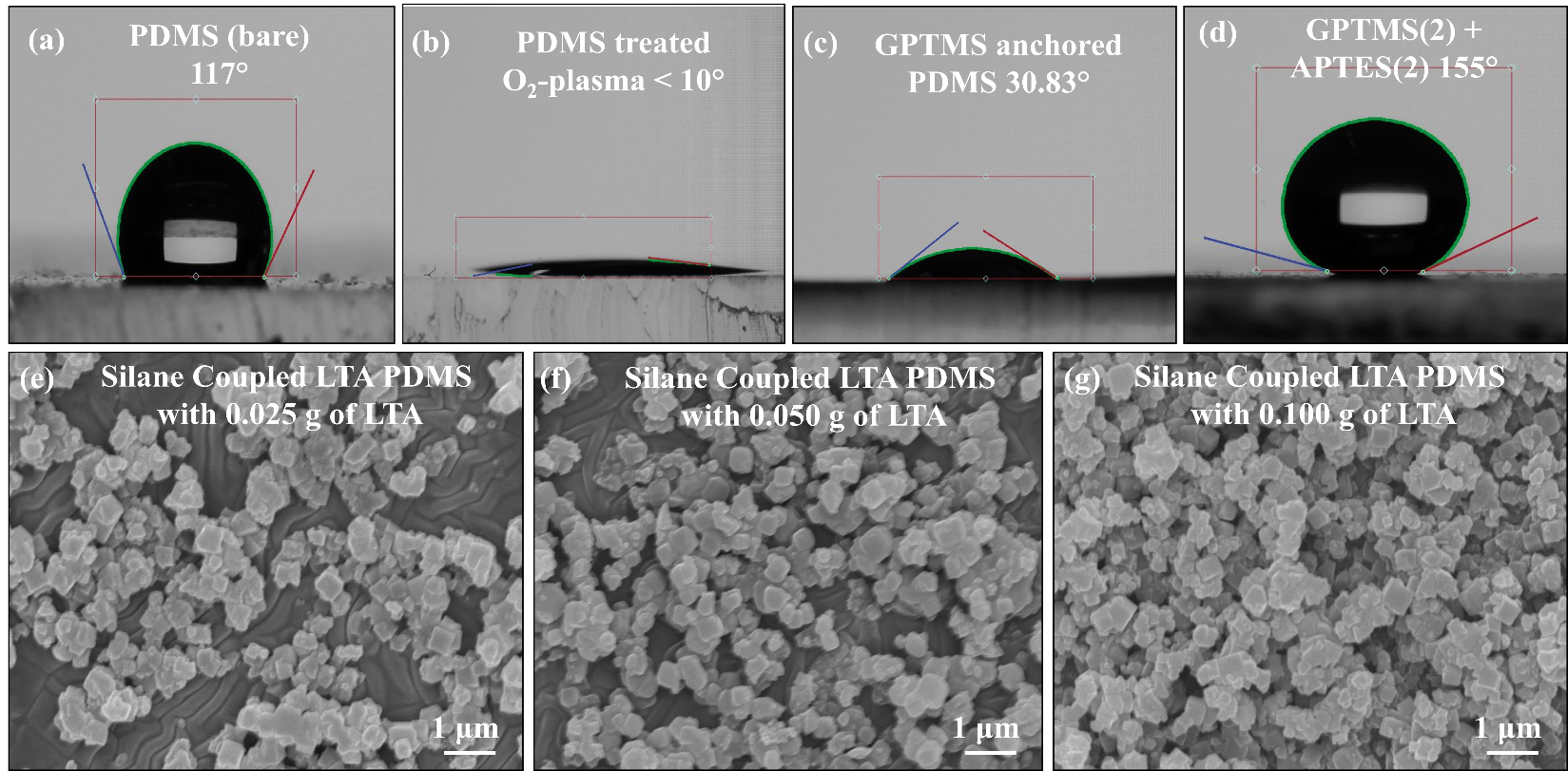
**

**Figure S3:** Contact angle measurement (a) PDMS (bare), (b) PDMS treated O_2_-plasma, (c) GPTMS anchored PDMS, (d) GPTMS (2mM) + APTES (2mM). Silane coupled on LTA PDMS with (e) 0.025 g, (f) 0.050 g, and (g) 0.10 g.

1. **EDX profile of as-synthesized LTA zeolite and APTES functionalized LTA zeolite.**


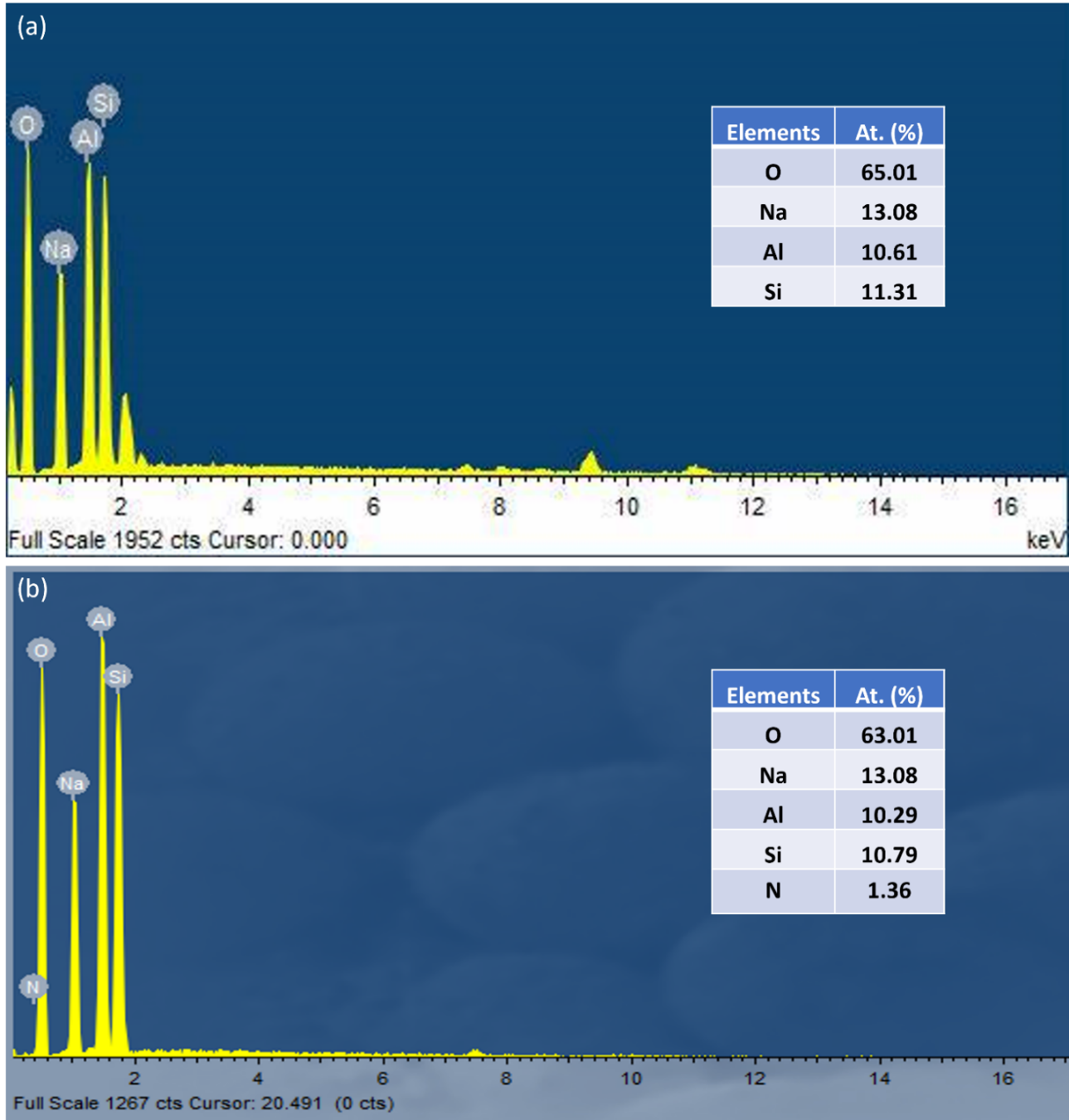


**Figure S4:** EDX profile for (a) as-synthesized LTA-zeolite and (b) APTES-functionalized LTA-zeolite.

1. **Electrical Characterization:**

In brief, silane-coupled LTA/PDMS was used as the electropositive layer and polyethene terephthalate (PET) was used as the electronegative layer. The PET sheet supports the triboelectric dielectric layers to press and release quickly. The linear damping system operates within a specified voltage range of 8 V, 12 V, 16 V, 20 V, and 24 V. It generates corresponding frequencies of 4 Hz, 8 Hz, 10 Hz, 12 Hz, and 14 Hz and generates a resultant force of 1 N, 2 N, 3 N, 4 N, and 5 N, respectively as shown in Supplementary Figure S5a-b. The damping force was measured using a DYHW-116 mini button load cell 10 kg compression force sensor connected with a DY220 load cell controller. The open circuit voltage was measured using a Rohde & Schwarz RTO 1014 oscilloscope at 1 GHz with 10 GSa/S using a 10 MΩ input impedance probe. Short circuit measurements were performed using a Keysight 4200A SCS source measurement unit. The 24V linear motor damping system was used to analyze the performance of TENG devices by varying the frequency, as shown in Supplementary Figure S5a-b. The temperature and humidity are controlled using a heating platform and humidifier, respectively, and the humidity level is monitored using the reference sensor HTU21D connected with Arduino UNO, and relative humidity change can be observed on an LCD display. The temperature is monitored using the TC 44 controller. The open circuit voltage and short circuit current are recorded in a closed chamber. The instantaneous power of the TENG was calculated by multiplying voltage and current by each load resistance value. Physiological monitoring using TENG is performed by attaching a device to a monitored part of the human body.

1. **Experiment Setup:**

**
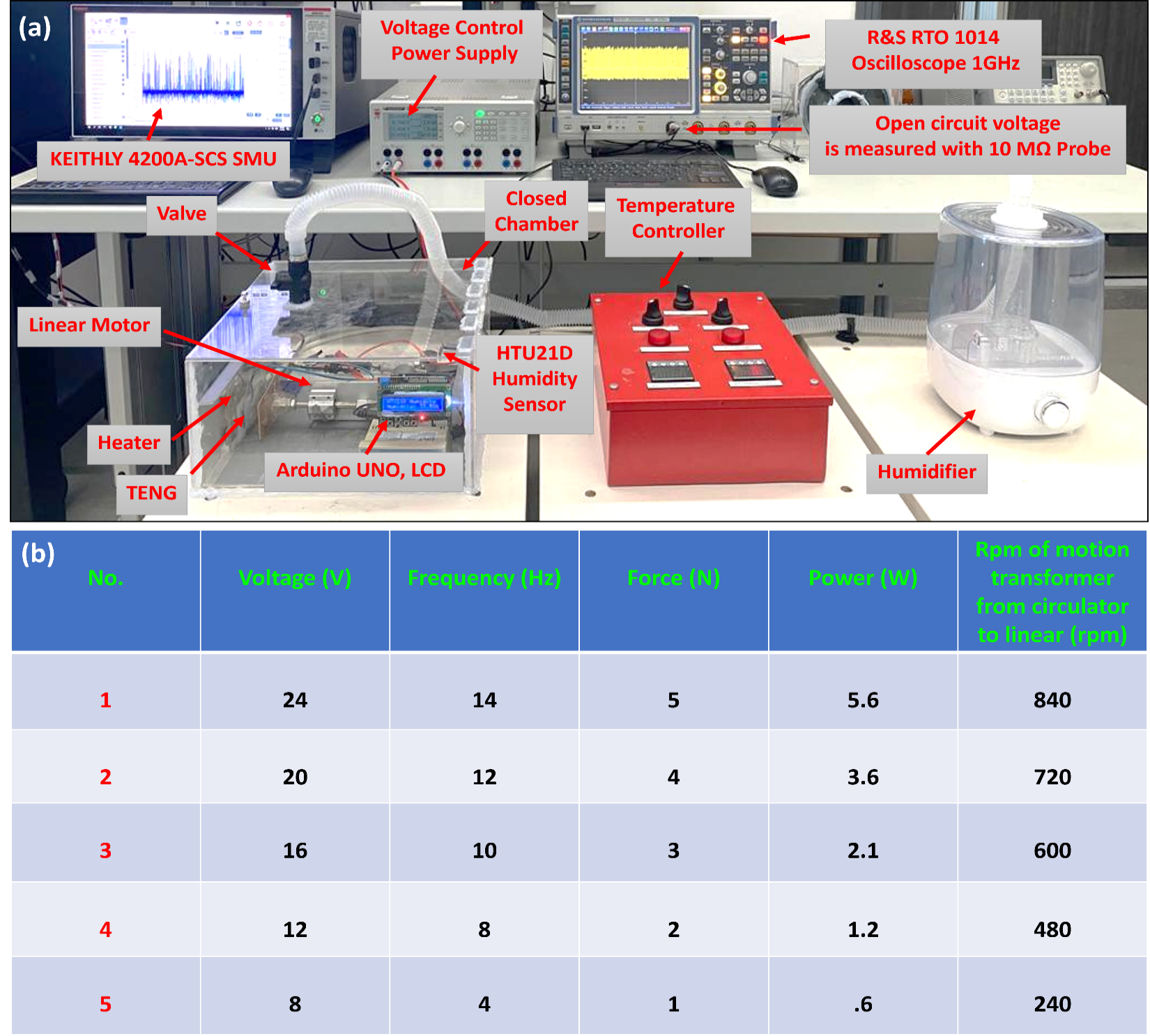
**

**Figure S5.** (a) Photograph of the linear damping system used for the characterization of TENG. (b) Controlling parameters of the linear damping system.

1. **Phase Analyses of Silane Coupled LTA /PDMS TENG**


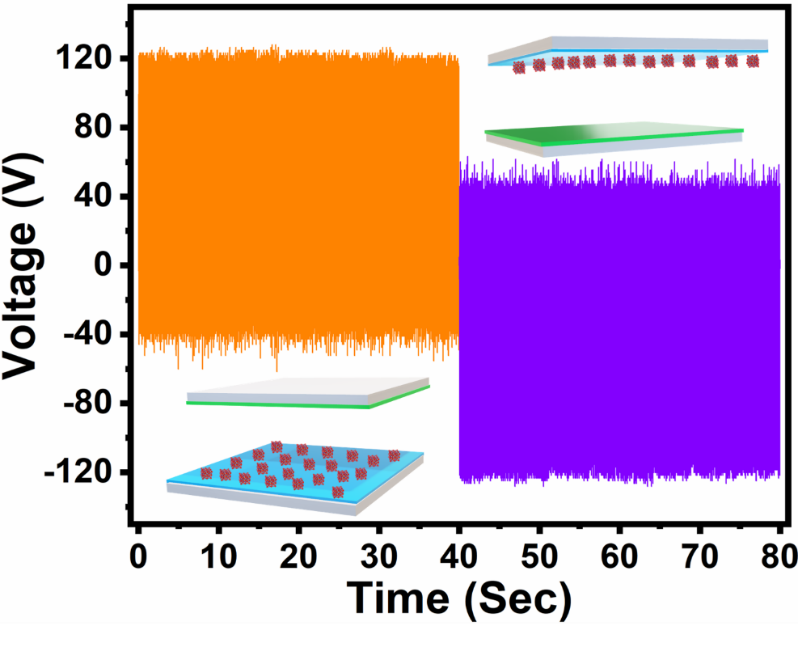


**Figure S6:** The phase response of the TENG device.

1. **Stacking device stability analysis of Silane Coupled LTA/PDMS TENG**


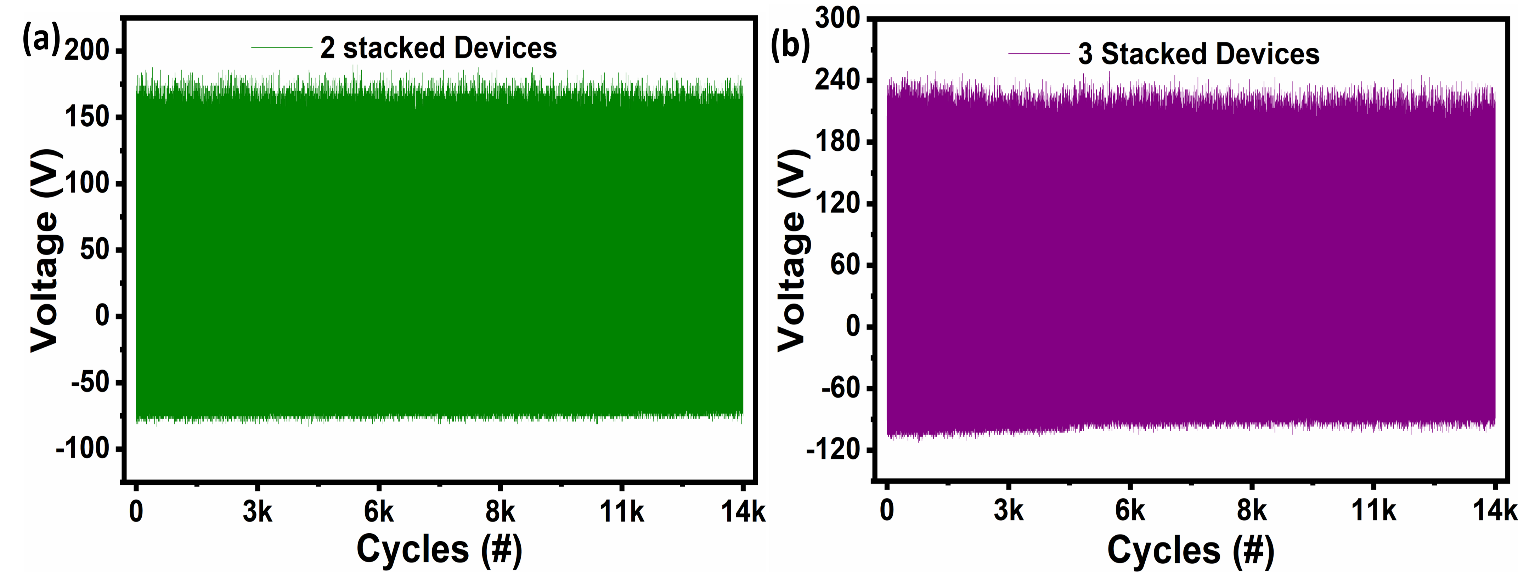


**Figure S7:** The TENG stability for more than 14,000 cycles using (a) 2 stacked and (b) 3 stacked units in series.

1. **Rectified Voltage of Silane Coupled LTA/PDMS TENG**


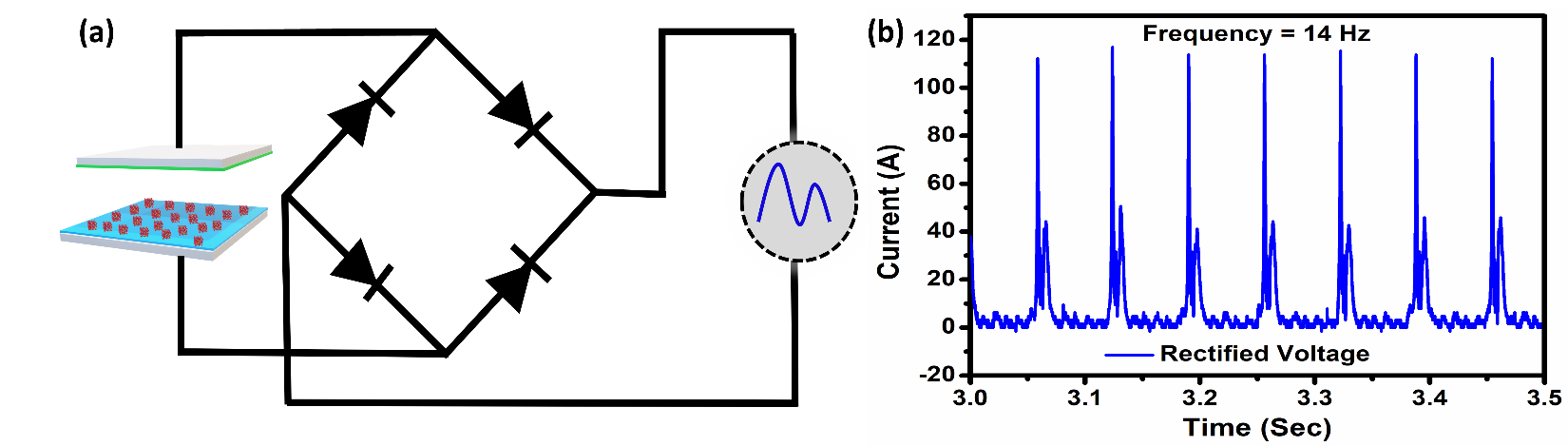


**Figure S8:** (a) Circuit for the rectified output voltage of TENG. (b) The detailed analysis of the rectified output voltage shows a rectified signal.

1. **Self-powered Electronics**

The LTA/PDMS TENG performance for energy storage was also analyzed for charging commercial capacitors of 4.7 µF and 10 µF units via a bridge rectifier circuit with simple hand-tapping force to operate microelectronic devices and for lighting LEDs. Figure S9a depicts the whole circuitry, which includes a bridge rectifier and a capacitor as supporting components. Figure S9a show that the charged capacitor was used to power low-power electronic devices (stopwatch and calculator). Given the stable output performance of the LTA/PDMS TENG, they can drive low-power energy devices without needing any energy storage units, as shown in Figure S9a. The complete circuit diagram to light up the LEDs is shown in Figure S9b, where a bridge rectifier is employed as a supporting device. The LTA/PDMS TENG was utilized to light up about 56 LEDs.


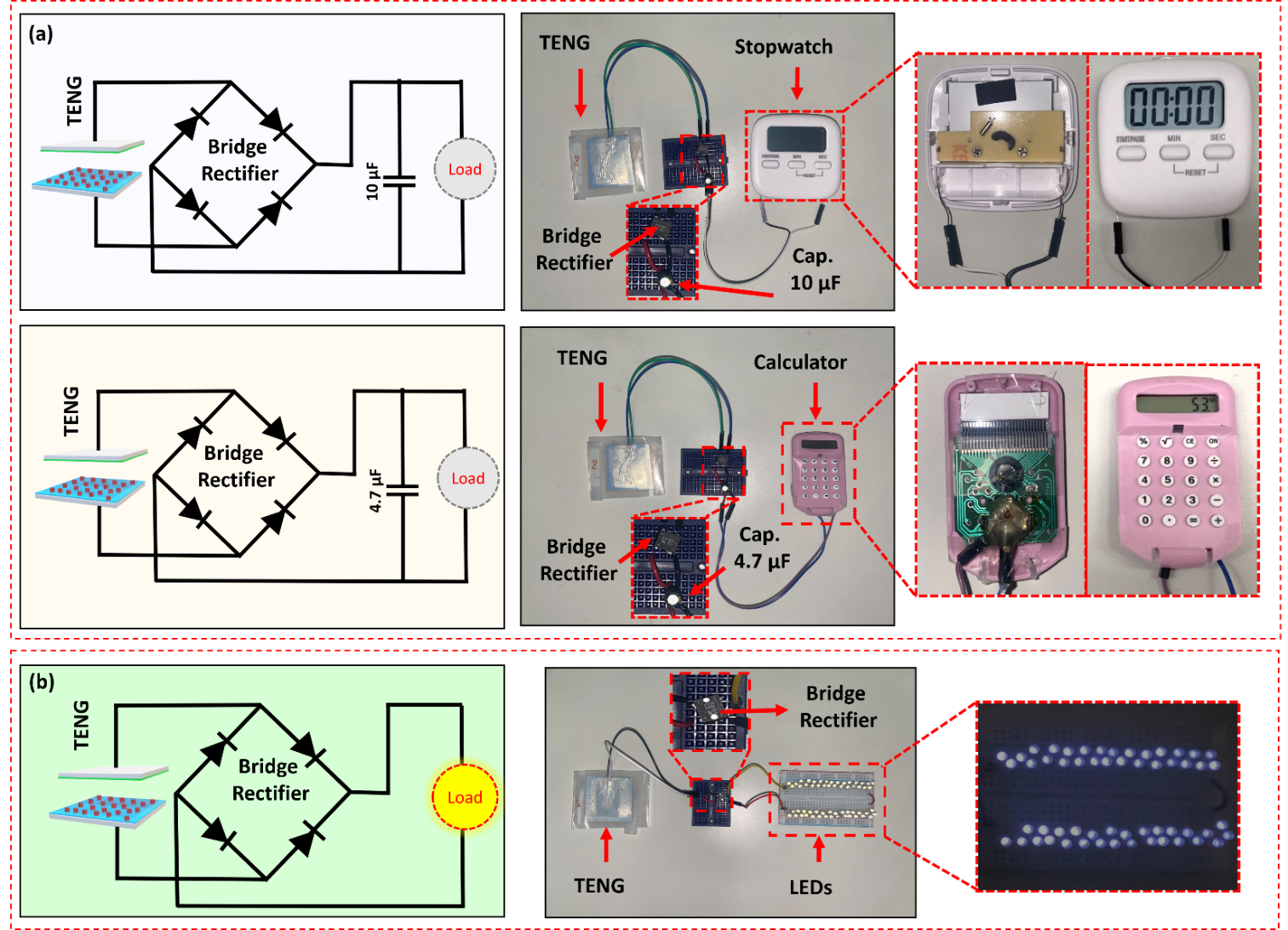


**Figure S9:** Practicality of TENG based on silane-coupled LTA/PDMS tribopositive layer used for powering low-power electronic devices. (a) The system diagram and realized image of the circuit diagram are used to power up low-power devices (stopwatch and calculator) using energy-storage units (capacitors), and a photograph shows the electronic stopwatch and calculator powering. (b) The system diagram and realized image of the circuit diagram are used to power up the LEDs directly using the bridge rectifying circuit, and the photograph of the 56 LEDs with stable luminance is taken when driven by the TENG.

1. **Performance Comparison**

The performance of LTA/PDMS TENG is compared to the reported work in the literature, as shown in Table S1. To summarize, the manufactured TENG offers enormous potential for energy-generating applications. Khandelwal et al.^3^ report TENG based on ZIF-7, ZIF-9, ZIF-11 and ZIF-12. The output, 60 V and 1.1 µA in vertical contact-separation mode with a surface area of 6.25 cm^2,^ is generated by the Al/ZIF-7/spacer/Kapton /Al TENG with a power density of 0.766 µW/cm^2^. In another study, Khandelwal et al. ^4^ report TENG with device structure Al/ZIF-62/spacer/Teflon/Al, with an active area of 6.25 cm^2^ that generates an output power density of 0.968 µW/cm^2^ and an open circuit voltage of 62 V and a short circuit current of 1.4 µA. Khandelwal et al. ^5^ also reported ZIF-8 TENG with ITO-coated PET/ZIF-8/spacer/Kapton/Cu device structure. The MOF-TENG operates in vertical contact-separation mode and produces a steady output of 164 V and 7 µA and a power density of 39.2 µW/cm^2^ with a device area of 6.25 cm^2^. Hajra et al. ^6^ reported Cu/Alpha Cyclodextrin MOF/Teflon/Cu TENG, with a device area of 4 cm^2^, produces an output voltage of 80 V, a short circuit current of 0.6 µA, and a power density of 8 µW/cm^2^. Khandelwal et al. ^7^ report Al/MIL-88A/FEP/Al TENG with a device area of 6.25 cm^2^. The device output produces an output of 88V, 2.2 µA, and a power density of 1.62 µW/cm^2^. Hajra et al.^8^ reported Cu/COF/Kapton/Cu with a device area of 6.25 cm^2^ produces an output voltage of 40 V and 0.4 µA and a power density of 0.848 µW/cm^2^. Hajra et al.^9^ reported the Cu/ZiF-67/Teflon/Cu device structure. The device produces an output voltage of 70 V and 1 µA and a power density of 15 µW/cm^2^. Gu et al. ^10^ report an antibacterial composite film based on Ag-zeolite polypropylene (PP) film composite for the fabrication of TENG. The TENG device produces an output voltage of 40 V and a short circuit current of 0.05 µA. In this work, LTA zeolite powder is directly applied to Al tape with device structure Al/LTA powder/spacer/Al, which results in output performance of 60 V, 7 µA, and power density of 16 µW/cm^2^ with an active area of 9 cm^2^. Further, this work reports Al/ LTA/PDMS/spacer/PET/Al TENG with improved performance. The proposed device shows high performance with an active area of 9 cm^2^, producing an output voltage of 120 V, a short circuit current of 15 µA, and a power density of 42.6 µW/cm^2^. To summarize, the manufactured TENG offers enormous potential for energy-generating applications and a new choice for highly robust and environmentally stable energy-harvesting tribo-materials.

**Table S1.** Literature summary of the electrical performance of previously reported tribo-electric nanogenerators and comparison to (LTA/PDMS)/PET TENG device from this work.

| TENG | Area (cm^2^) | Voltage  (V) | Current (µA) | Power density  (µW/cm^2^) | Ref. |
| --- | --- | --- | --- | --- | --- |
| ZIF-7 / KAPTON | 6.25 | 60 | 1.1 | 0.776 | ^3^ |
| ZIF-62 / Teflon | 6.25 | 62 | 1.4 | 0.968 | ^4^ |
| ZIF-8/Kapton | 6.25 | 164 | 7 | 39.2 | ^5^ |
| Cyclodextrin MOF/Teflon | 4 | 80 | 0.6 | 8 | ^6^ |
| MIL-88A / FEP | 6.25 | 88 | 2.2 | 1.62 | ^7^ |
| COF/Kapton | 6.25 | 40 | 0.4 | 0.848 | ^8^ |
| ZIF-67/Teflon | ---- | 70 | 1 | 15 | ^9^ |
| Ag-Zeolite: Polypropylene | 4 | 40 | 0.05 | ---- | ^10^ |
| **LTA powder/PET** | **9** | **60** | **7** | **16** | **This work** |
| **Silane-coupled LTA/PDMS/PET** | **9** | **120** | **15** | **42.6** | **This work** |

**References**

1 Melitz, W., Shen, J., Kummel, A. C. & Lee, S. Kelvin probe force microscopy and its application. *Surface Science Reports* **66**, 1-27, doi:<https://doi.org/10.1016/j.surfrep.2010.10.001> (2011).

2 Michael, N. & Bhushan, B. Hierarchical roughness makes superhydrophobic states stable. *Microelectronic Engineering* **84**, 382-386, doi:<https://doi.org/10.1016/j.mee.2006.10.054> (2007).

3 Khandelwal, G., Maria Joseph Raj, N. P. & Kim, S.-J. Zeolitic Imidazole Framework: Metal–Organic Framework Subfamily Members for Triboelectric Nanogenerators. *Advanced Functional Materials* **30**, 1910162, doi:<https://doi.org/10.1002/adfm.201910162> (2020).

4 Khandelwal, G., Maria Joseph Raj, N. P. & Kim, S.-J. ZIF-62: a mixed linker metal–organic framework for triboelectric nanogenerators. *Journal of Materials Chemistry A* **8**, 17817-17825, doi:10.1039/D0TA05067A (2020).

5 Khandelwal, G., Chandrasekhar, A., Maria Joseph Raj, N. P. & Kim, S.-J. Metal–Organic Framework: A Novel Material for Triboelectric Nanogenerator–Based Self-Powered Sensors and Systems. *Advanced Energy Materials* **9**, 1803581, doi:<https://doi.org/10.1002/aenm.201803581> (2019).

6 Hajra, S. *et al.* A Green Metal–Organic Framework-Cyclodextrin MOF: A Novel Multifunctional Material Based Triboelectric Nanogenerator for Highly Efficient Mechanical Energy Harvesting. *Advanced Functional Materials* **31**, 2101829, doi:<https://doi.org/10.1002/adfm.202101829> (2021).

7 Khandelwal, G., Maria Joseph Raj, N. P., Vivekananthan, V. & Kim, S.-J. Biodegradable metal-organic framework MIL-88A for triboelectric nanogenerator. *iScience* **24**, 102064, doi:<https://doi.org/10.1016/j.isci.2021.102064> (2021).

8 Hajra, S. *et al.* Triazine skeletal covalent organic frameworks: A versatile highly positive surface potential triboelectric layer for energy harvesting and self-powered applications. *Nano Energy* **101**, 107620, doi:<https://doi.org/10.1016/j.nanoen.2022.107620> (2022).

9 Hajra, S. *et al.* A new insight into the ZIF-67 based triboelectric nanogenerator for self-powered robot object recognition. *Journal of Materials Chemistry C* **9**, 17319-17330, doi:10.1039/D1TC04729A (2021).

10 Gu, G. Q. *et al.* Antibacterial Composite Film-Based Triboelectric Nanogenerator for Harvesting Walking Energy. *ACS Applied Materials & Interfaces* **9**, 11882-11888, doi:10.1021/acsami.7b00230 (2017).
